# *Streptococcus pneumoniae* Serine Protease HtrA, but Not SFP or PrtA, Is a Major Virulence Factor in Pneumonia

**DOI:** 10.1371/journal.pone.0080062

**Published:** 2013-11-11

**Authors:** Sacha F. de Stoppelaar, Hester J. Bootsma, Aldert Zomer, Joris J. T. H. Roelofs, Peter W. M. Hermans, Cornelis van ’t Veer, Tom van der Poll

**Affiliations:** 1 Center for Infection and Immunity Amsterdam (CINIMA), Academic Medical Center, University of Amsterdam, Amsterdam, The Netherlands; 2 Center for Experimental and Molecular Medicine (CEMM), Academic Medical Center, University of Amsterdam, Amsterdam, The Netherlands; 3 Laboratory of Pediatric Infectious Diseases, Radboud University Medical Center, Nijmegen, The Netherlands; 4 Center for Molecular and Biomolecular Informatics, Radboud University Medical Center, Nijmegen, The Netherlands; 5 Department of Pathology, Academic Medical Center, University of Amsterdam, Amsterdam, The Netherlands; 6 Division of Infectious Diseases, Academic Medical Center, University of Amsterdam, Amsterdam, The Netherlands; Louisiana State University, United States of America

## Abstract

*Streptococcus (S.) pneumoniae* is a common causative pathogen in pneumonia. Serine protease orthologs expressed by a variety of bacteria have been found of importance for virulence. Previous studies have identified two serine proteases in *S. pneumoniae,* HtrA (high-temperature requirement A) and PrtA (cell wall-associated serine protease A), that contributed to virulence in models of pneumonia and intraperitoneal infection respectively. We here sought to identify additional *S. pneumoniae* serine proteases and determine their role in virulence. The *S. pneumoniae* D39 genome contains five putative serine proteases, of which HtrA, Subtilase Family Protein (SFP) and PrtA were selected for insertional mutagenesis because they are predicted to be secreted and surface exposed. Mutant D39 strains lacking serine proteases were constructed by in-frame insertion deletion mutagenesis. Pneumonia was induced by intranasal infection of mice with wild-type or mutant D39. After high dose infection, only D39Δ*htrA* showed reduced virulence, as reflected by strongly reduced bacterial loads, diminished dissemination and decreased lung inflammation. D39Δ*prtA* induced significantly less lung inflammation together with smaller infiltrated lung surface, but without influencing bacterial loads. After low dose infection, D39Δ*htrA* again showed strongly reduced bacterial loads; notably, pneumococcal burdens were also modestly lower in lungs after infection with D39Δ*sfp*. These data confirm the important role for HtrA in *S. pneumoniae* virulence. PrtA contributes to lung damage in high dose pneumonia; it does not however contribute to bacterial outgrowth in pneumococcal pneumonia. SFP may facilitate *S. pneumoniae* growth after low dose infection.

## Introduction

The bacterium *Streptococcus (S.) pneumoniae* is a major global cause of human disease [1]. *S. pneumoniae* is the most frequent cause of community-acquired pneumonia and a common pathogen in sepsis, the incidence being greatest at the extremes of age and in immune compromised individuals [2]. Although the discovery of antibiotics and the development of vaccines have reduced the health burden associated with pneumococcal infections, *S. pneumoniae* still causes over 2 million deaths annually [1] and increased bacterial resistance against currently available antibiotics could make pneumococcal infections an even larger health threat in the future [3]. Consequently, additional knowledge about this bacterium and its virulence factors is of importance.

In 1991, Courtney [4] was the first to describe a role for serine proteases as virulence factors for *S. pneumoniae*. Since then, serine protease orthologs have been found in many bacteria and numerous roles in virulence and pathogenesis have been described [5]–[16]. One of these serine proteases is HtrA (high-temperature requirement A), which has been identified as a virulence factor in several bacterial species. In *S. pneumoniae*, HtrA is important for bacterial stress response and protein quality control, and has a role in competence [11], [14], [17]. As a posttranslational regulator, HtrA is involved in bacteriocin activity and cell division [8], [15], [16]. HtrA has been identified as an important virulence factor for *S. pneumoniae*, *i.e*. HtrA deficient pneumococci demonstrated a dramatically reduced virulence in models of pneumonia and bacteremia [11]. Another investigation identified PrtA (cell wall-associated serine protease A) as a pneumococcal serine protease important for virulence after intraperitoneal infection [5].

In this study, we searched for additional serine proteases as virulence factors of *S. pneumoniae*. By reannotating the *S. pneumoniae* D39 genome using a subsystems approach [18] and by screening all proteins for the presence of serine protease associated domains using Interproscan [19], we identified SFP (subtilase family protein) as an additional surface-exposed pneumococcal serine protease besides HrtA and PrtA, and generated directed gene knockout mutants of *htrA* (SPD_2068), *sfp* (SPD_1753) and *prtA* (SPD_0558) in *S. pneumoniae* strain D39. We tested their virulence in an *in vivo* pneumonia model by inoculating mice with viable wild-type (WT) and mutant *S. pneumoniae* via the airways, and compared several outcome parameters 48h after infection.

## Materials and Methods

### Serine protease search

All proteins encoded in the genome of *S. pneumoniae* D39 where screened for the presence of serine protease associated domains using Interproscan [19]. Domains IPR009003 and IPR001940 identified HtrA, domain IPR008357 identified SFP, while domains IPR000209 and IPR015500 identified both SFP and PrtA as predicted to encode serine proteinases. An additional search for proteins predicted to have serine protease activity was performed by examining membership of GO category 0008236, resulting in the identification of SPD_1765 and SPD_1920. Blast analysis of the proteome of *S. pneumoniae* D39 with HtrA, SFP and PrtA with an E-value cut-off of 0.1 showed limited protein sequence similarity of SFP with PrtA, but no other putative serine proteases were identified. Furthermore the *S. pneumoniae* D39 genome was re-annotated using the +6RAST subsystems approach [18] and the resulting annotations where searched for protease encoding proteins. No other obvious serine proteases could be detected using these methods. Subcellular localization prediction of the putative serine proteases was performed with SignalP [20].

### Construction of directed deletion mutants

Directed-deletion mutants of *S. pneumoniae* D39 lacking HtrA (D39Δ*htrA*), SFP (D39Δ*sfp*) or PrtA (D39Δ*prtA*) were generated by allelic exchange of the target gene with a spectinomycin resistance marker essentially as described previously [21]. Briefly, an extension PCR was performed to join 400–500 bp 5’ and 3’ flanking sequences of the target gene with the spectinomycin resistance cassette (obtained from pR412T7). The resulting PCR products were introduced by competent stimulating peptide (CSP-1)-induced transformation into D39. Transformants were selected on the basis of spectinomycin resistance and were checked by PCR for recombination at the desired location on the chromosome. Subsequently, the D39 WT strain was transformed with 1 μg chromosomal DNA isolated from the mutants to prevent the accumulation of inadvertent mutations elsewhere on the chromosome. At the same time, D39 was mock-transformed to obtain a coupled WT strain. All primers used in this study are shown in Table 1.

**Table 1:** Oligonucleotide primers used in this study.

Primer name	Target ^a, b^	Sequence (5'- 3')
Primers for generation of directed mutants		
PBpR412_L	Spec^R^ cassette	GCCGCTCTAGAACTAGTGG
PBpR412_R	Spec^R^ cassette	GATACCCCTCGAATTGACGC
PBTnMr9	Spec^R^ cassette control primer	CAATGGTTCAGATACGACGAC
SPD_2068_L1	*htrA* left flanking region	TTCCCTTCAATGGCTAACAC
SPD_2068_L2	*htrA* left flanking region	CCACTAGTTCTAGAGCGGCAAACTACCCAAGGCTCCAC
SPD_2068_R1	*htrA* right flanking region	GACTTGCCCATCTTATCTGC
SPD_2068_R2	*htrA* right flanking region	GCGTCAATTCGAGGGGTATCACCATTCTATCGGAGACACC
SPD_2068_C	*htrA* gene control primer	TCGCTGAGAATCTGTGTCAG
SPD_1753_L1	*sfp* left flanking region	CCTCTTGGATTAGAGACAATC
SPD_1753_L2	*sfp* left flanking region	CCACTAGTTCTAGAGCGGCTTTCGCTAGTCTGAGTGTG
SPD_1753_R1	*sfp* right flanking region	TAGTTGTGGTAACCTGTTTGC
SPD_1753_R2	*sfp* right flanking region	GCGTCAATTCGAGGGGTATCATTACAAGTCAGTTTAGTTG
SPD_1753_C	*sfp* gene control primer	CTTTCGCCTCCACCAGTAAC
SPD_0558_L1	*prtA* left flanking region	TTCAAACCACGTCAACGTCG
SPD_0558_L2	*prtA* left flanking region	CCACTAGTTCTAGAGCGGCTGCAGCTGTAGTTAGTGAC
SPD_0558_R1-2	*prtA* right flanking region	TAACCGTCCAATAGACTTCG
SPD_0558_R2	*prtA* right flanking region	GCGTCAATTCGAGGGGTATCAGCCCAGACACTATTAGCTG
SPD_0558_C	*prtA* gene control primer	GTGTCTGCTAAGACTACCTC

### In vitro growth assay

Mid-log growing mutant or WT *S. pneumoniae* were diluted to an optical density (OD) of 0.1 (620 nm wavelength) in Todd Hewitt broth (Oxoid microbiology products, Thermo Scientific, Hampshire, UK) with 0.5% Yeast extract (THY). Cultures were incubated at 37°C in a 5.0% CO_2_ incubator. OD was measured every hour for the next 5 hours.

### Animals

Specific pathogen-free C57BL/6 male and female mice were purchased from Harlan Sprague-Dawley (Horst, the Netherlands). Experimental groups were age- and sex matched, and housed in the Animal Research Institute Amsterdam under standard care. All experiments were conducted with mice between 10 and 12 weeks of age.

### Ethics statement

This study was carried out in concordance with the ‘Wet op de Dierproeven’ in the Netherlands. The Institutional Animal Care and Use Committee of the Academic Medical Center approved all experiments. All efforts were made to minimize suffering. Induction of pneumonia happened under isoflurane anaesthesia.

### Experimental study design

Pneumonia was induced by intranasal inoculation with *S. pneumoniae* D39, D39Δ*htrA*, D39Δ*sfp* or D39Δ*prtA* (serotype 2; 5×10^5^ or 5×10^4^ colony forming units (CFU) in 50 µL isotonic saline) using previously described methods [22]–[24]. Mice were euthanized 48 hours after induction of pneumonia (N  =  8 mice per group). Blood was obtained from the inferior vena cava and diluted 4:1 with citrate. Bronchoalveolar lavage fluid (BALF), lung, spleen and liver were harvested as described [22]–[24] and organs were homogenised in five volumes of sterile isotonic saline. The left lung lobe was fixed in 10% buffered formalin and embedded in paraffin. Total cell numbers in BALF were determined by an automated cell counter (Coulter Counter, Coulter Electronics, Hialeah, FL, USA). Differential cell counts were performed on cytospin preparations stained with a modified Giemsa stain (Diff-Quick; Dade Behring AG, Düdingen, Switzerland). For bacterial quantification blood, BALF, and organ homogenates were serially diluted by 10-fold in sterile isotonic saline and plated onto sheep-blood agar plates. Following 16 hours of incubation at 37°C CFU were counted. For further measurements, homogenates were diluted 1:1 with lysis buffer (300 mM NaCl, 30 mM Tris, 2 mM MgCl_2_, 2 mM CaCl_2_, 1% (v/v) Triton X-100, pH 7.4) with protease inhibitor mix and incubated for 30 minutes on ice, followed by centrifugation at 680 g for 10 minutes. Supernatants were stored at –20°C until analysis.

### Histopathology

Four-micrometer sections of the left lung lobe were stained with hematoxylin and eosin (H&E). Slides were coded and scored by a pathologist blinded for group identity for the following parameters: interstitial inflammation, endothelialitis, bronchitis, oedema, pleuritis and presence of thrombi. All parameters were rated separately from 0 (condition absent) to 4 (most severe condition). The total histopathological score was expressed as the sum of the scores of the individual parameters, with a maximum of 24. In addition, pulmonary infiltrate was scored as a percentage of total lung surface occupied by confluent infiltrates.

### Assays

Interleukin (IL)-6, tumor necrosis factor alpha (TNF-α), keratinocyte-derived cytokine (KC) and IL-1β were measured using commercially available ELISA kits (R&D Systems, Abingdon, UK). Myeloperoxidase (MPO; Hycult, Uden, the Netherlands) was measured by ELISA according to manufacturers’ instructions.

### Statistical analysis

Data are expressed as box and whisker plots showing the smallest observation, lower quartile, median, upper quartile and largest observation, or as medians with interquartile ranges. Comparisons between groups were first performed using Kruskal-Wallis one-way analysis of variance test; in case of significant differences, differences between groups were tested using the Mann-Whitney *U* test. All analyses were done using GraphPad Prism version 5.01 (GraphPad Software, San Diego, CA). P-values < 0.05 were considered statistically significant.

## Results

### *S. pneumoniae* D39 genome contains three putative serine proteases

**Figure 1:**
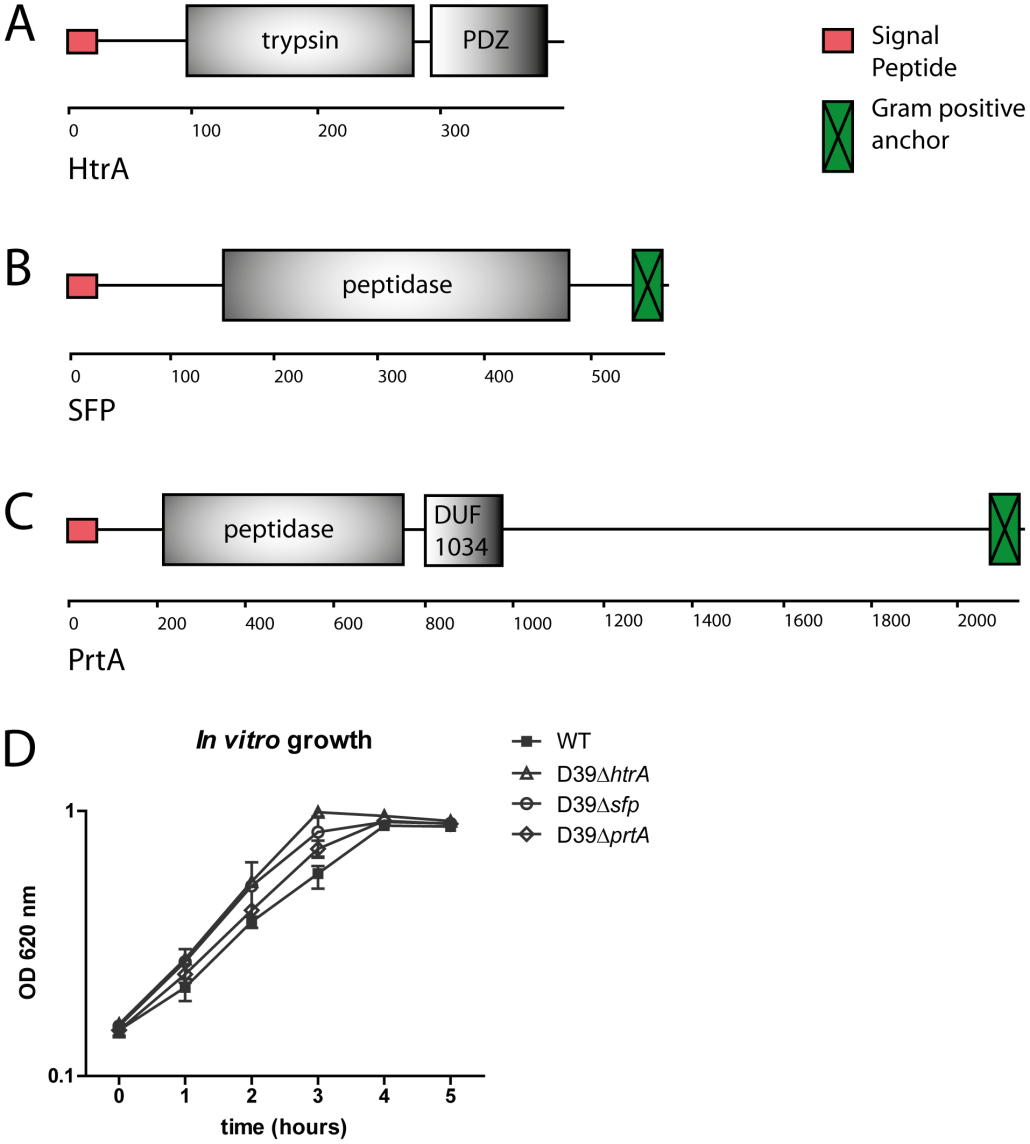
HtrA, SFP and PrtA: structure and *in vitro* growth of mutant *S. pneumoniae*. Structure of serine protease HtrA (A), SFP (B) and PrtA (C), predicted by SMART PROTEIN online sequence analysis (http://smart.embl-heidelberg.de/). Number of amino acids is indicated underneath each enzyme. The predicted PDZ (Post synaptic density protein, Drosophila disc large tumor suppressor, and Zonula occludens-1 protein) domain in HtrA could be involved in protein-protein interactions. The DUF (Domain of Unknown Function) 1034 domain in PrtA is predicted to have serine-type endopeptidase activity. (D) Mutant *S. pneumoniae* strains did not show reduced growth compared to WT *S. pneumoniae in vitro*.

To identify all serine protease proteins encoded in the genome of *S. pneumoniae* D39, we screened the D39 proteome for the presence of serine protease domains using Interproscan as described in the Experimental procedures. Furthermore, the D39 genome was re-annotated to identify additional proteases. This led to the identification of several proteases, of which only HtrA (SPD_2068), PrtA (SPD_558) and SFP (SPD_1753) were predicted to have both serine protease activity (figure 1A-C) and be secreted and surface-exposed based on the presence of a signal sequence [20]. The predicted PDZ (Post synaptic density protein, Drosophila disc large tumor suppressor, and Zonula occludens-1 protein) domain in HtrA could be involved in protein-protein interactions. The DUF (Domain of Unknown Function) 1034 domain in PrtA is predicted to have serine-type endopeptidase activity. Two additional putative serine proteases were identified by their membership of GO category 0008236 (SPD_1765 and SPD_1920), but they were excluded from further analysis because they did not contain a signal sequence or a cell surface exposed serine protease domain. BLAST analysis of the D39 proteome with HtrA, PrtA and SFP did not reveal any additional putative serine protease encoding genes.

### *S. pneumoniae* D39ΔhtrA, but not D39Δsfp or D39ΔprtA, displays diminished growth and dissemination *in vivo*

**Figure 2:**
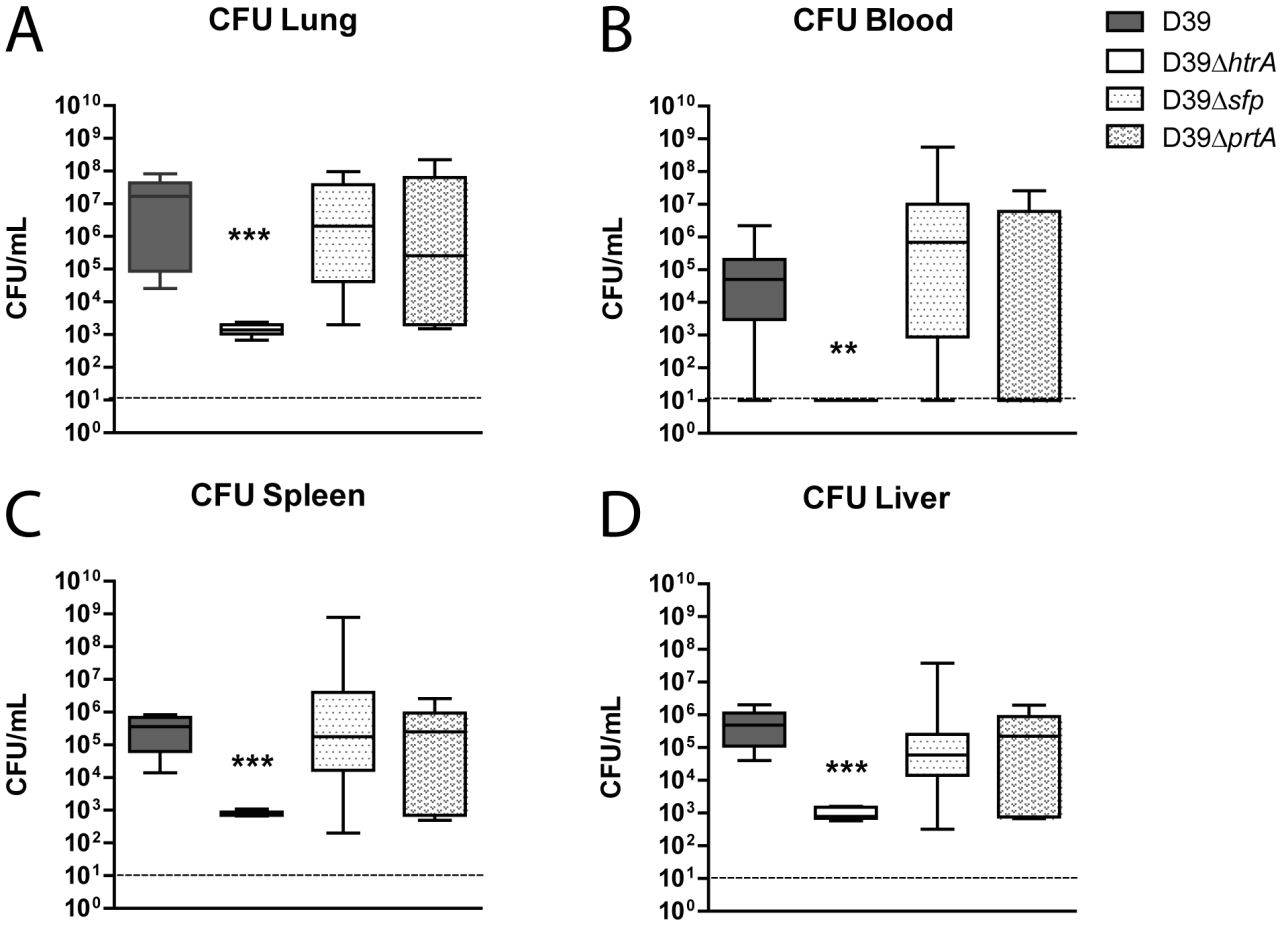
*S. pneumoniae* D39Δ*htrA*, but not D39Δ*sfp* or D39Δ*prtA*, displays diminished growth and dissemination *in vivo*. Mice were infected with WT or mutant *S. pneumoniae* (5×10^5^ CFU) via the intranasal route and euthanized 48 hours later. Bacterial counts were determined in lung (A), blood (B), spleen (C) and liver (D). Data are expressed as box- and whisker plots depicting the smallest observation, lower quartile, median, upper quartile and largest observation. N  =  8 mice per group at each time point. *** P < 0.005 versus WT *S. pneumoniae*.

Mutant *S. pneumoniae* strains did not show reduced growth compared to WT *S. pneumoniae* in THY at 37 °C and 5.0% CO_2_ (figure 1D). To study the role of the three serine proteases in *S. pneumoniae* virulence, we infected mice with 5×10^5^ CFU of either WT D39, D39Δ*htrA*, D39Δ*sfp* or D39Δ*prtA* via the airways and quantified bacterial loads at the primary site of infection (lungs) and distant body sites (blood, spleen and liver) 48 hours later (figure 2). WT *S. pneumoniae* D39, D39Δ*sfp* and D39Δ*prtA* had multiplied in the lungs, accompanied by dissemination to spleen and liver; the counts of these three pneumococcal strains were similar in all body sites examined. In contrast, D39Δ*htrA* counts were markedly lower in the lungs and in all distant body sites when compared with the other three strains (P < 0.0005). In addition, no bacteria could be detected in the blood of D39Δ*htrA* infected animals (P < 0.005).

### Reduced lung inflammation during pneumonia caused by *S. pneumoniae* D39ΔhtrA or D39ΔprtA

**Figure 3:**
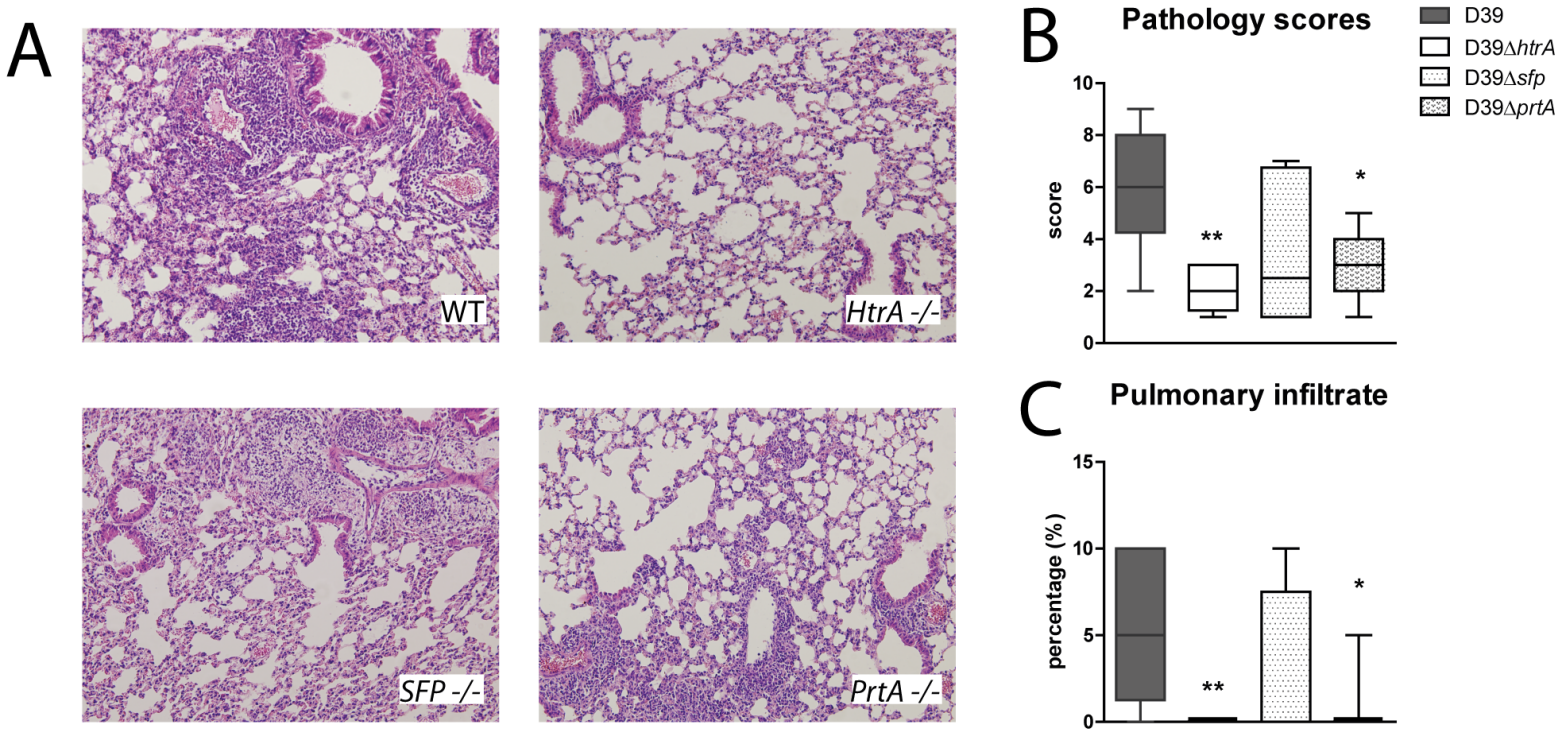
Reduced lung inflammation during pneumonia caused by *S. pneumoniae* D39Δ*htrA* and D39Δ*prtA*. Mice were infected with WT or mutant *S. pneumoniae* (5×10^5^ CFU) via the intranasal route and euthanized 48 hours later. (A) Representative microphotographs of H&E stained lung sections of WT or mutant *S. pneumoniae* infected mice (10 times original magnification). (B) Total lung histopathology scores expressed as box- and whisker plots depicting the smallest observation, lower quartile, median, upper quartile and largest observation. (C) Pulmonary infiltrate as percentage of total lung surface. No infiltrates were observed in the D39Δ*htrA* infected group; only one mouse with an infiltrate was observed in the D39Δ*prtA infected group.* N  =  8 mice per group at each time point. * P < 0.05, ** P < 0.01 versus WT *S. pneumoniae*.

**Figure 4:**
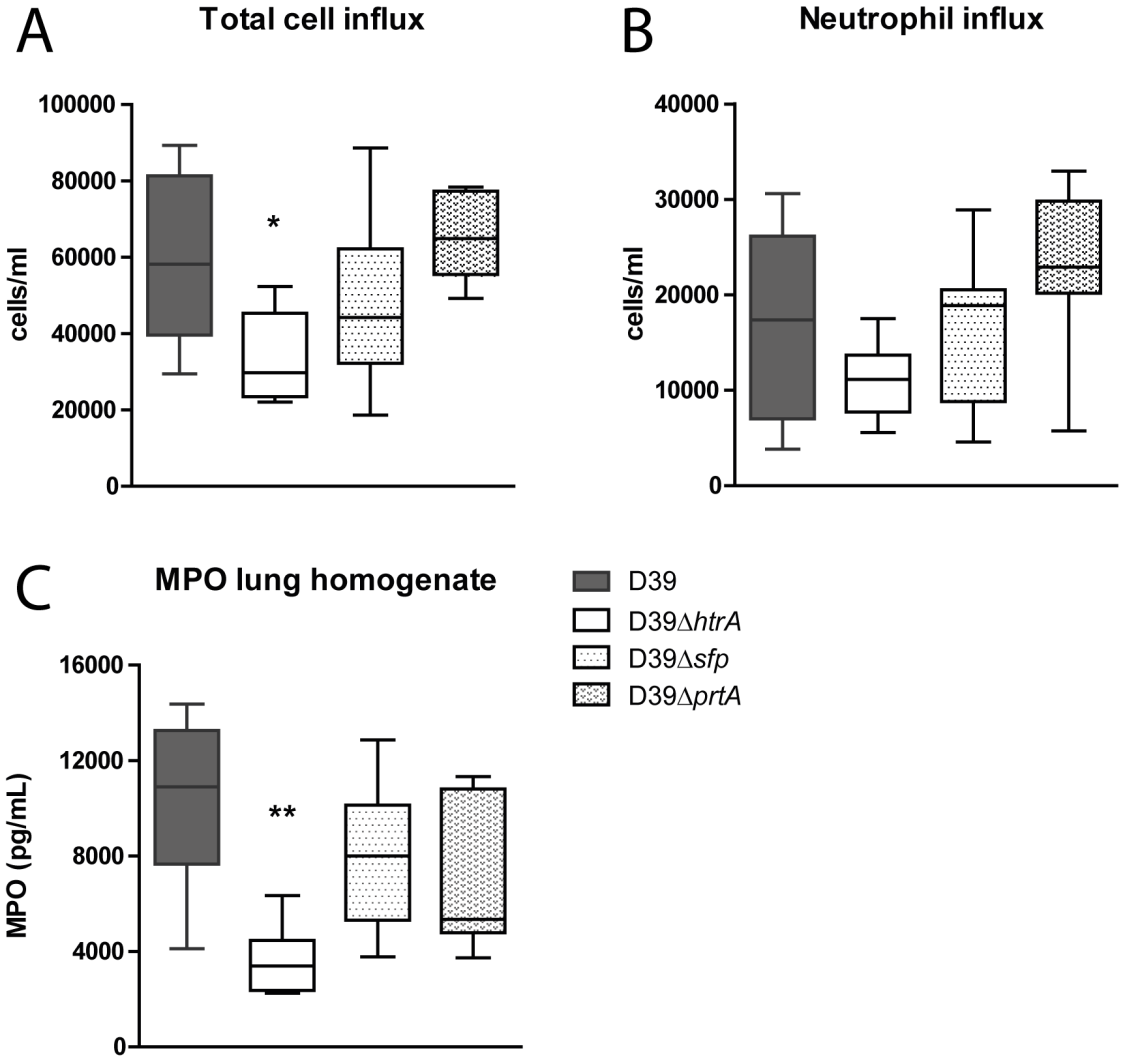
Diminished cell and neutrophil influx during pneumonia caused by *S. pneumoniae* D39Δ*htrA*. Mice were infected with WT or mutant *S. pneumoniae* (5×10^5^ CFU) via the intranasal route and euthanized 48 hours later. Cell (A) and neutrophil (B) influx were determined on BALF cytospin preparations. As a marker of neutrophil influx in lung tissue, MPO was measured in whole lung homogenates (C). Data are expressed as box- and whisker plots depicting the smallest observation, lower quartile, median, upper quartile and largest observation. N  =  8 mice per group at each time point. * P < 0.05 versus WT *S. pneumoniae*.

This model of pneumococcal pneumonia is associated with histological features in the lung characteristic for lower respiratory tract infection, including interstitial inflammation, endothelialitis, edema, inflammatory infiltrates and pleuritis [25]. To determine the impact of the three *S. pneumoniae* serine proteases on the induction of these inflammatory alterations, we semi-quantitatively scored lung histology slides after pneumonia caused by WT D39, D39Δ*htrA*, D39Δ*sfp* or D39Δ*prtA* (figure 3). The lungs of D39Δ*htrA* infected mice displayed significantly less inflammation and a smaller infiltrated lung surface (P < 0.01). Remarkably, in spite of similar bacterial loads, the lungs of D39Δ*prtA* infected mice also displayed lower histopathology scores when compared with lungs of WT D39 infected mice, together with a smaller infiltrated lung surface (P < 0.05). Considering that neutrophils play a key role in the inflammatory response during respiratory tract infection by *S. pneumoniae* [1], [26], we next determined total cell and neutrophil counts in BALF after infection with WT D39, D39Δ*htrA*, D39Δ*sfp* or D39Δ*prtA* (figure 4). We additionally measured MPO concentration in whole lung homogenates, as a marker of neutrophil content of lung tissue. Total cell counts were lower in BALF obtained from D39Δ*htrA* infected mice (P < 0.05); a similar trend was observed for neutrophil influx. In accordance, whole lung MPO concentrations were lower after infection with D39Δ*htrA* (P < 0.05). The deletion of either *sfp* or *prtA* did not influence cell recruitment. Cytokines and chemokines play an eminent role in the regulation of inflammation during pneumonia [1], [26]. Therefore, we measured cytokines (TNFα, IL-1β, IL-6) and a chemokine (KC) in whole lung homogenates as an additional readout for pulmonary inflammation. Lung IL-1β, IL-6 and KC levels were lower in D39Δ*htrA* when compared with the other three *S. pneumoniae* strains (table 2).

**Table 2:** Lung cytokine and chemokine levels.

	*S. pneumoniae*	D39	D39Δ*htrA*	D39Δ*sfp*	D39Δ*prtA*
Lung homogenate, t = 48h (pg/mL)					
	IL-6	1689 (257 – 2864)	174 (136 – 220) **	1076 (220 – 3045)	260 (174 – 3717)
	TNF-α	498 (216 – 857)	220 (189 – 277)	592 (229 – 1290)	285 (171 – 805)
	KC	7164 (747 – 8697)	362 (322 – 444) **	3218 (659 – 7962)	482 (300 – 8355)
	IL-1β	724 (116 – 1117)	81 (73 – 97) **	363 (129 – 859)	106 (79 – 1047)

Mice were infected with the *S. pneumoniae* strain indicated (5×10^5^ CFU) via the intranasal route and euthanized 48 hours later. Data are expressed as medians and interquartile range of 7 or 8 mice per group. ** P < 0.01 versus WT *S. pneumoniae*.

### Modest role for SFP after low dose infection

**Figure 5:**
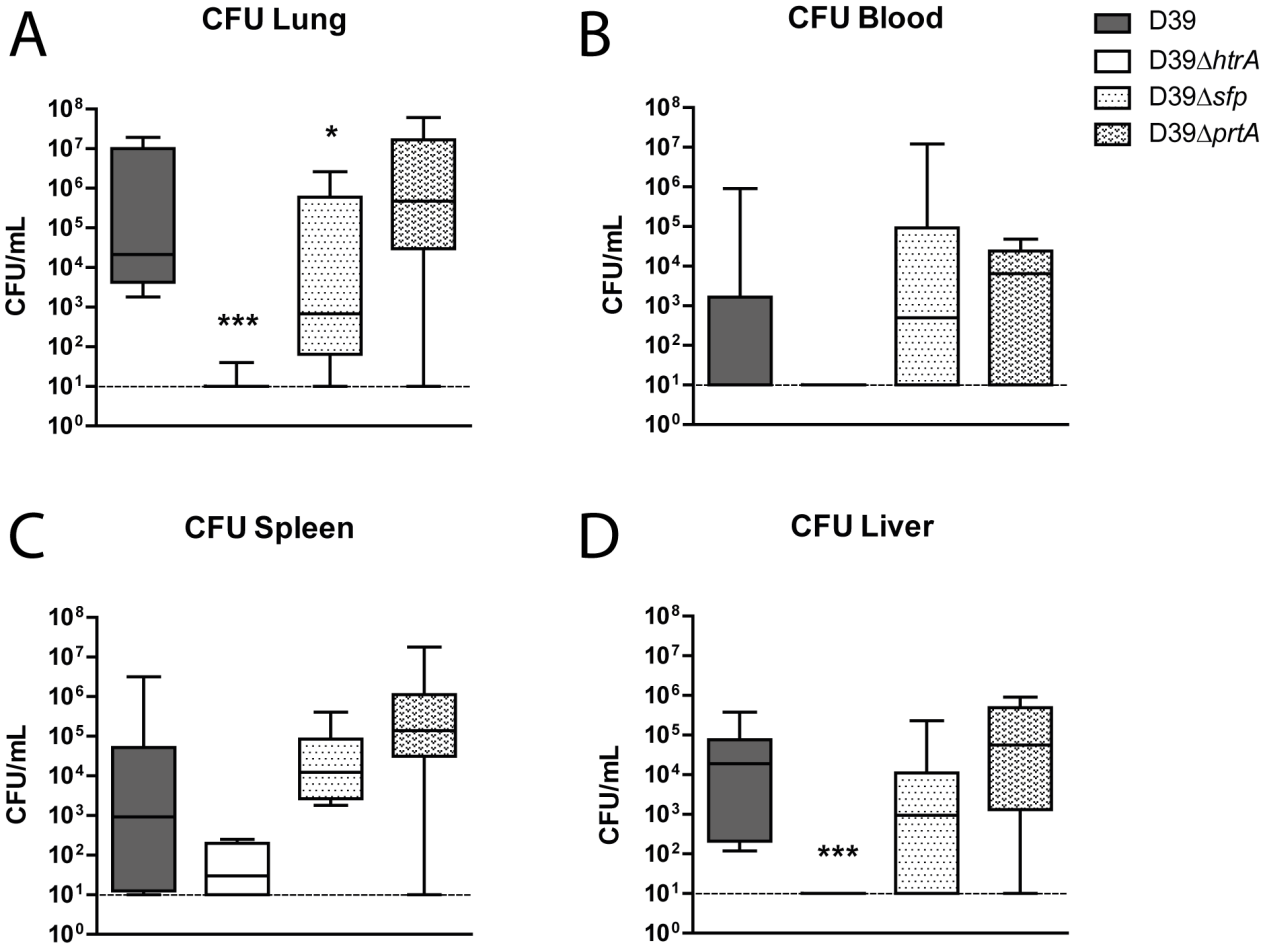
HtrA strongly contributes to virulence after low dose infection with a more modest role for SFP. Mice were infected with a 10-fold lower inoculum of WT or mutant *S. pneumoniae* (relative to the infectious dose used in the experiments shown in figures 2–4; 5×10^4^ CFU) via the intranasal route and euthanized 48 hours later. Bacterial counts were determined in lung (A), blood (B), spleen (C) and liver (D). Data are expressed as box- and whisker plots depicting the smallest observation, lower quartile, median, upper quartile and largest observation. N  =  8 mice per group at each time point. * P <0.05, ** P < 0.01, *** P < 0.005 versus WT *S. pneumoniae*.

**Figure 6:**
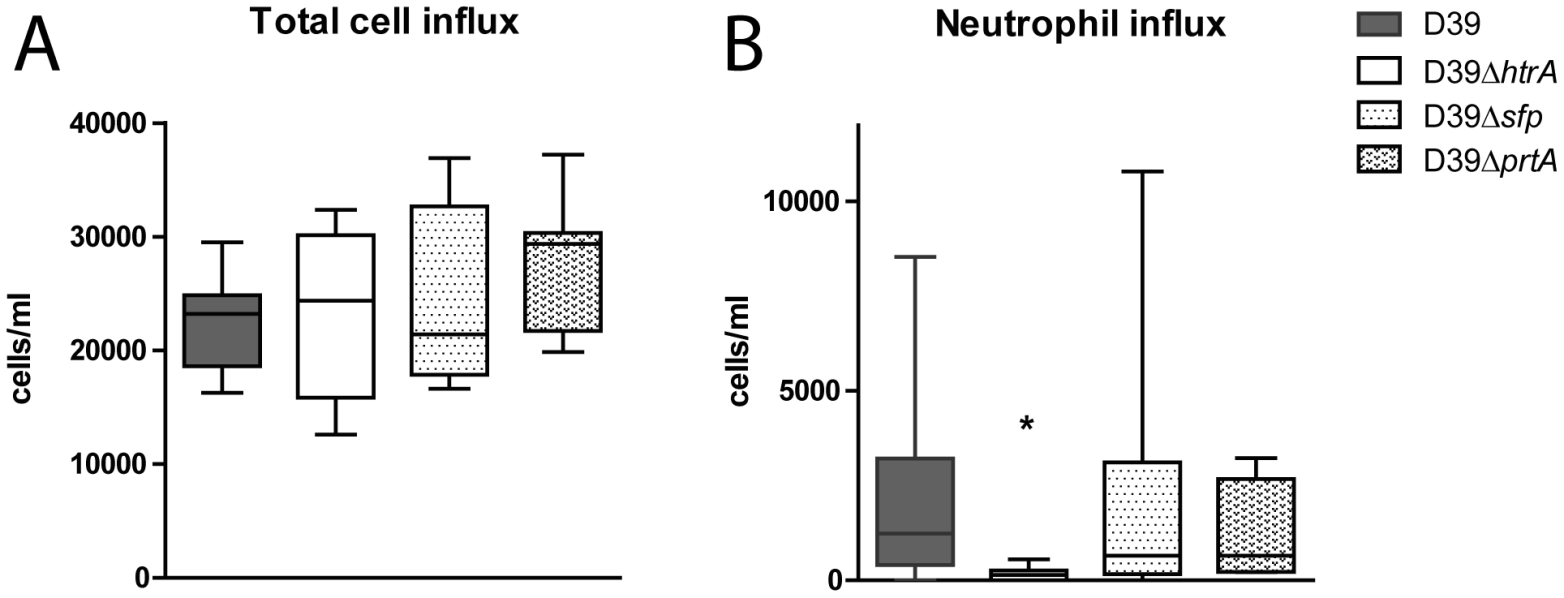
Diminished cell and neutrophil influx during pneumonia caused by low dose *S. pneumoniae* D39Δ*htrA*. Mice were infected with a 10-fold lower inoculum of WT or mutant *S. pneumoniae* (relative to the infectious dose used in the experiments shown in figures 2–4; 5×10^4^ CFU) via the intranasal route and euthanized 48 hours later. Cell (A) and neutrophil (B) influx were determined on BALF cytospin preparations. Data are expressed as box- and whisker plots depicting the smallest observation, lower quartile, median, upper quartile and largest observation. N  =  8 mice per group at each time point. * P < 0.05 versus WT *S. pneumoniae*.

The data presented above confirm that HtrA plays a significant role in *S. pneumoniae* virulence [11]. We argued that the contribution of PrtA and SFP to *S. pneumoniae* virulence could be more subtle and as such not noticed in our model with a relatively high bacterial dose. Accordingly, we repeated our infection experiments with a 10-fold lower inoculum (5×10^4^ CFU). These studies again revealed the reduced virulence of D39Δ*htrA*, as reflected by lower bacterial loads in lungs (P < 0.005) and liver (P < 0.005) at 48 hours post infection (figure 5). Of interest, pneumococcal burdens were also modestly but significantly lower in lungs after infection with D39Δ*sfp* (P < 0.05); this difference with WT D39 was not observed in blood or distant organs. Cell influx in low dose pneumonia was modest (Figure 6), with less than half of the total cell counts in BALF compared to high dose infection. No difference in total cell numbers was observed between WT or mutant *S. pneumoniae* infected mice (figure 6A). Neutrophil influx determined by cell differentiation on cytospin slides however was diminished in D39Δ*htrA* infected animals (figure 6B) (P < 0.05).

## Discussion

Serine protease orthologs have been found in many bacteria, contributing to their virulence to a significant extent [4]-[14]. Previous research identified the serine protease HtrA as a major virulence factor in *S. pneumoniae* during experimentally induced pneumonia [11], [14]. We here sought for additional *S. pneumoniae* serine proteases and determined their role in virulence during respiratory tract infection *in vivo*. Our main findings are that the *S. pneumoniae* D39 genome expresses three putative secreted, surface-exposed serine proteases: HtrA, SFP and PrtA. We confirmed reduced virulence after high and low dose infection of D39Δ*htrA* [11], [14], as reflected by strongly reduced bacterial loads, diminished systemic dissemination and decreased lung inflammation. After high dose infection D39Δ*prtA* induced significantly less lung inflammation without influencing bacterial loads. Pneumococcal burdens were also modestly but significantly lower in lungs after low dose infection with D39Δ*sfp*. These data reveal two additional pneumococcal serine proteases that can modify the host response during pneumonia, albeit clearly to a more modest extent than HtrA.

Structure and function of HtrA have been widely studied. *S. pneumoniae* HtrA has been identified to help bacteria survive environmental pressures such as elevated temperature, oxidative stress, and osmotic stress [11]. Another study revealed an important role for HtrA during *S. pneumoniae* cell division [15]. HtrA additionally has a distinct role in bacteriocin activity by reducing pneumocin expression [8], [16]. Pneumocins mediate intra- and interspecies competition *in vitro* and have been shown to provide a competitive advantage *in vivo* [16]. In the present study, we confirmed the strongly reduced virulence of D39Δ*htrA* in pneumonia [11]. D39Δ*htrA* infected animals displayed more than 3 logs lower bacterial counts in their lungs at 48 hours after infection; in addition, their lungs showed minimal signs of lower respiratory tract infection upon histopathological examination. There was significantly less dissemination to distant organs in D39Δ*htrA* infected mice. Notably, D39Δ*htrA* could be detected in spleen and liver, although blood cultures were sterile in all experiments. Ibrahim et al [11] concluded that D39Δ*htrA S. pneumoniae* did not disseminate from the lungs based on negative blood cultures; our data, however, indicate that D39Δ*htrA* is able to spread to distant body sites.

It is known that apolactoferrin can kill many species of bacteria, including *Streptococcus pneumoniae*. Lactoferricin, an N-terminal peptide of apolactoferrin, and fragments of it are even more bactericidal than apolactoferrin. PrtA cleaves apolactoferrin, greatly enhancing the killing activity of apolactoferrin and its cleavage products [13]. Thus, in theory, PrtA deficiency might cause increased rather than decreased virulence due to a diminished capacity of apolactoferrin to kill *S. pneumoniae*. Nonetheless, the only *in vivo* study performed thus far with PrtA deficient *S. pneumoniae* showed decreased virulence compared to WT *S. pneumoniae* when injected intraperitoneally in mice [5]. Our pneumonia model is more relevant to determine the role of PrtA in *S. pneumoniae* virulence, as pneumonia is the primary illness caused by pneumococci. In both high and low dose infection, bacterial outgrowth, pulmonary cell influx and cytokine release were similar after induction of pneumonia with D39Δ*prtA* or WT *S. pneumoniae*. Interestingly, histopathology scores of lungs and the percentage of infiltrated lung surface were significantly lower in D39Δ*prtA* infected animals than in mice inoculated with WT *S. pneumoniae*, suggesting that PrtA has a modest role in the induction of pulmonary inflammation during pneumococcal pneumonia without influencing cellular influx or cytokine release in the lungs, and bacterial multiplication and dissemination.

To our knowledge, the role of SFP in pneumococcal virulence has not been studied before. SFP has homology with *S. agalactiae* CspA, a serine protease capable of inactivating chemokines *in vitro* [7]. While after high dose infection the growth of D39Δ*sfp* was indistinguishable from that of WT *S. pneumoniae*, this mutant strain demonstrated slightly reduced growth in the lungs in low dose pneumonia. This modest role of SFP mediated virulence should be confirmed in additional studies using different *S. pneumoniae* strains.

We identified two additional hypothetical proteins, SPD_1765 and SPD_1920, predicted to have serine protease activity. These were not examined further here as their serine protease domain was likely not exposed to the outer cell surface. It may be of interest to examine the roles of intracellular serine protease activity for SPD_1765 and SPD_1920 in *S. pneumoniae* virulence in future research.

The current investigation addressed the role of three serine proteases expressed by *S. pneumoniae* (HtrA, SFP and PrtA) in virulence *in vivo* by comparing bacterial growth and dissemination and the accompanying inflammatory response in the lung after 48 hours of airway infection by newly generated deletion mutants. This time point was selected since our main endpoint of interest was bacterial growth; the 48-hour time point reflects late stage pneumonia shortly before mice are expected to die. Our data confirm the previously reported important role for HtrA in *S. pneumoniae* virulence [11]. In this previous paper, reconstitution of HtrA into D39ΔhtrA reverted D39ΔhtrA to its full virulence [11]. We here created a new HtrA deletion mutant and our investigation is limited by the fact that we did not reconstitute HtrA in our independently generated D39ΔhtrA strain. Of note, however, our main objective was to identify *new* pneumococcal serine proteases as virulence factors in pneumonia. Since our results only reveal a significant role for the already established HrtA [11], we think that the absence of a HrtA reconstitution experiment does not jeopardize our main conclusion (*i.e.*, that the other proteases identified do not significantly contribute to the virulence of *S. pneumoniae*). In contrast to its reported role in intraperitoneal infection [5] we here show that PrtA does not influence bacterial multiplication and dissemination in pneumococcal pneumonia, however, PrtA had a modest role in the induction of pulmonary inflammation. Finally, in the first studies reported to date, we provide evidence that SFP may facilitate *S. pneumoniae* growth after low dose infection of the lower respiratory tract, although clearly these data need to be confirmed in independent experiments involving more time points. Together these data firmly establish that virulence of *S. pneumoniae* is dominated by HtrA.
